# Interactome analysis brings splicing into focus

**DOI:** 10.1186/s13059-015-0707-0

**Published:** 2015-07-07

**Authors:** Daniel Dominguez, Christopher B. Burge

**Affiliations:** Department of Biology, Massachusetts Institute of Technology, Cambridge, MA 02142 USA; Department of Biological Engineering, Massachusetts Institute of Technology, Cambridge, MA 02142 USA

## Abstract

The spliceosome is a huge molecular machine that assembles dynamically onto its pre-mRNA substrates. A new study based on interactome analysis provides clues about how splicing-regulatory proteins modulate assembly of the spliceosome to either activate or repress splicing.

Please see related Research article: http://www.genomebiology.com/2015/16/1/119/abstract

## Introduction

Massive molecular machines comprising dozens to hundreds of proteins, as well as RNA components, catalyze transcription, RNA splicing and translation. These machines vary substantially in their modes of assembly and regulation. While ribosomes come preassembled (in prokaryotes) or in just two pieces (eukaryotes), the spliceosome that catalyzes removal of introns from eukaryotic primary transcripts comprises several pieces that must assemble de novo on each of its intronic substrates. Once assembled, the spliceosome is one of the largest complexes in the cell, comprising hundreds of proteins linked by uncounted stable interactions, as well as transient interactions that form as rearrangements occur during splicing [1]. These pieces include five small nuclear ribonucleoprotein particles (snRNPs), each formed from a distinct small nuclear RNA (snRNA) wrapped in a shell of proteins. These snRNPs, as well as other proteins, comprise the catalytic core of the spliceosome.

Dozens of protein factors can regulate splicing, mediating alternative splicing outcomes, such as exon skipping, inclusion or intron retention, to produce distinct mRNA and protein isoforms from a single gene locus [1, 2]. These factors can be roughly grouped into splicing activators, splicing repressors and factors with more-variable activities, but the mechanisms by which activation and repression occur are still not well understood. Akerman and colleagues [3] report the generation of a spliceosome-centric interactome to identify specific features of splicing activators and repressors that might help uncover regulatory mechanisms.

## Modeling the splicing interactome

The major spliceosome, which is responsible for splicing the vast majority of introns, contains approximately 150 to 300 proteins, depending on its stage of assembly and other variables. Some of these proteins form highly stable complexes, such as the rings formed by Sm proteins, whereas others interact only transiently at specific stages of spliceosome assembly, making the splicing interactome particularly complex.

**Fig. 1:**
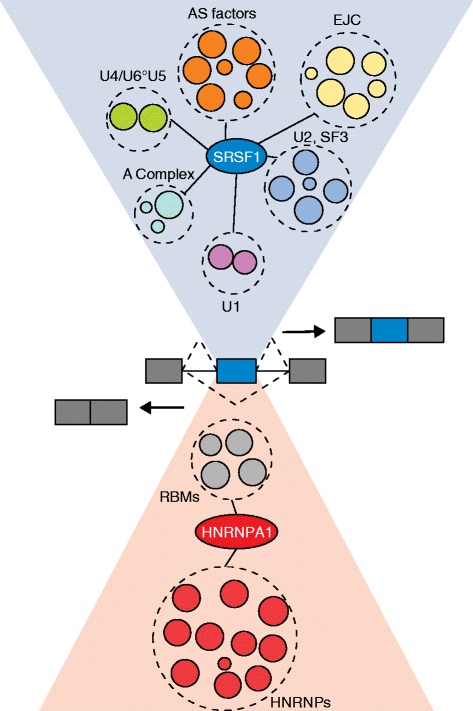
Representation of the interactomes of a prototypical splicing activator, SRSF1, and a prototypical splicing repressor, HNRNPA1. Akerman and colleagues [3] report that activators — which promote exon inclusion (shown at right) — form more interactions with components of the spliceosome than repressors, which promote exon exclusion (*left*). *EJC* exon junction complex; *hnRNP* heterogeneous nuclear ribonucleoprotein; *RBM* RNA binding motif; *SRSF* serine/arginine-rich splicing factor

In an effort to make sense of this complexity and to understand the functions of splicing regulators, Akerman and colleagues [3] build a probabilistic model of the protein–protein interactions (PPIs) among splicing factors called the ‘probabilistic spliceosome’ (or PS-network) (Fig. 1). They use the Human Protein Reference Database (HPRD) as a source of data on interactions, which is based mostly on large-scale yeast two-hybrid (Y2H) experiments. Central to their analysis is the graph theory concept of ‘transitivity’, or clustering coefficient, which measures the extent to which a pair of nodes in a network share interactions with other nodes. For example, Y2H might have failed to detect an interaction between two splicing factors ‘A’ and ‘B’ but successfully detected that each interacts with several of the same spliceosomal proteins, yielding a high transitivity score that enables the inference that A and B are often in proximity and likely interact. The authors also use gene-expression data, following the logic that interacting proteins should tend to be coexpressed. The Y2H and expression data were combined in a Bayesian approach to produce a composite ‘probability of interaction’ (P_in_) for each pair of proteins, and PS-networks were built from interactions predicted at each of several P_in_ cutoffs. These networks were then tested on an independently generated Y2H interaction matrix screen of splicing factors that detected over 600 interactions between approximately 200 proteins [4]. The results were promising, detecting 55 % of spliceosome interactions at a moderate threshold (P_in_ ≥ 0.1) that retained a high prediction specificity of 85 %.

The resulting PS-network identifies approximately ten clusters of proteins based on similarity in interaction profiles, using an approach similar to that of Ravasz and colleagues [5]. Many of these clusters correspond to established functional groupings of factors, with a cluster of U1/U2 snRNP factors, a cluster associated with the B^act^ and C spliceosomal complexes, and a cluster that included SR proteins and heterogeneous nuclear ribonucleoproteins (hnRNPs). These clusters could provide insights into the predominant stage of splicing at which a particular factor acts. One could also compare these data with those of clusters of splicing factors identified by Papasaikas et al. [6], who used an orthogonal, functional approach involving clustering based on similarity in the pattern of splicing changes observed following depletion of spliceosomal and splicing regulatory factors through RNA interference (RNAi). The apparent similarities between these clusterings support the intuitive notion that interacting proteins tend to function similarly in splicing regulation.

## Implications for the regulation of splicing

Whether an intron is spliced ultimately comes down to whether a catalytically active spliceosome is assembled on it or not. Regulation of splicing can involve either recruiting components of the splicing machinery to particular locations or blocking their access, or it can occur by modulating interactions between core splicing components, such as snRNPs. The Akerman study focuses on two canonical classes of splicing factors — SR proteins and hnRNPs — both of which are widely expressed and well conserved across metazoans. Most SR proteins (serine/arginine-rich splicing factors SRSF1–SRSF7) can activate inclusion of exons to which they bind at sites known as exonic splicing enhancers, whereas SRSF9–SRSF11 mostly act as splicing repressors. The hnRNPs also include several factors that act mostly as activators, and others such as hnRNPs A1 and A2B1 that function primarily as repressors, based on a recent comprehensive genomic analysis of binding and regulation [7]. A number of studies have explored the mechanisms by which activators and repressors function, supporting a variety of models. These include models in which activators recruit snRNPs to nearby sites or promote exon-bridging interactions between snRNPs, and models in which repressors bind on both sides of and ‘loop out’ exons or inhibit interactions between snRNPs [8]. However, the detailed mechanisms involved in the regulation of splicing remain poorly understood.

Akerman and colleagues observe that splicing activator proteins and splicing repressors exhibit very different patterns of interactions in the PS-network. Activators such as SRSF1 have high predicted connectivity to multiple subsets of spliceosomal factors, whereas repressors such as HNRNPA1 interact with fewer factors. For example, SRSF1 was predicted to interact with components of all five snRNPs, including with factors of the core Sf3 family of U2 snRNP, whereas HNRNPA1 was predicted to interact mostly with other hnRNP proteins. Furthermore, the SRSF1 interactome included many more direct and RNA-independent PPIs, whereas the PPIs of HNRNPA1 were more often RNA dependent.

The authors followed up these observations by performing immunoprecipitation-mass-spectrometry, confirming many of the interactions identified by the PS-network, including a novel interaction between SRSF1 and specific Sf3a subunits. Direct interactions between SRSF1 and the Sf3a complex support a recruitment model, whereby SR proteins facilitate the assembly of the U2 snRNP (or stabilize its binding) on the pre-mRNA to which they are bound. Previous biochemical experiments have shown that SRSF1 is present in U2-containing cellular fractions and stabilizes the U2 snRNP on pre-mRNA in in vitro splicing assays [9].

A steric-hindrance model for hnRNP-mediated repression only requires that hnRNPs block the accessibility of core splicing machinery to pre-mRNA. While hnRNPs are not particularly bulky proteins, their known ability to bind cooperatively and spread on or loop out pre-mRNA molecules could help to explain how hnRNP binding to relatively short sequence motifs with moderate affinity can result in blockade of spliceosome assembly [10]. Akerman and colleagues also identify direct interactions between splicing activators and repressors (e.g., SRSF1 with SRSF9 and SRSF10). Such interactions might help to explain why canonical activators can sometimes repress, and canonical repressors can sometimes activate.

## Conclusions

An important question for future study is to characterize the interactomes and understand regulation by the dozens of other splicing regulators that are not SR proteins or hnRNPs. These factors can often activate or repress splicing with similar frequency, usually in a manner that depends on where they bind in relation to the regulated exons, often described by an ‘RNA map’. For example, several factors might activate exon inclusion when bound downstream of the exon, but might repress splicing when bound upstream.

Ultimately, one hopes that our understanding of the splicing interactome will be unified with our understanding of the structures and regulatory functions of individual factors and complexes in order to fully illuminate the underlying mechanisms.

## Abbreviations

hnRNP: Heterogeneous nuclear ribonucleoprotein
HPRD: Human Protein Reference Database
P_in_: Probability of interaction
PPI: Protein–protein interaction
RNAi: RNA interference
snRNA: Small nuclear RNA
snRNP: Small nuclear ribonucleoprotein particle
SRSF: Serine/arginine-rich splicing factor
Y2H: Yeast two-hybrid
